# Flavonoid compound breviscapine suppresses human osteosarcoma Saos‐2 progression property and induces apoptosis by regulating mitochondria‐dependent pathway

**DOI:** 10.1002/jbt.22633

**Published:** 2020-09-24

**Authors:** Zhijun Wang, Hongyan Li, Jiyuan Yan, Yang Liu

**Affiliations:** ^1^ Department of Orthopedics Renmin Hospital of Qingyang Qingyang China; ^2^ Lanzhou Vocational Technical College Lanzhou China; ^3^ Department of Orthopedics, Tongji Hospital, Tongji Medical College Huazhong University of Science and Technology Wuhan China

**Keywords:** breviscapine, mitochondrial apoptosis, osteosarcoma

## Abstract

This study was aimed to investigate the ability of a flavonoid compound breviscapine (BVP) to suppress growth and elicit apoptosis in human osteosarcoma (OS) Saos‐2 cells. The cells were cultured in vitro and treated with three concentrations of BVP (80, 160, and 320 μg/ml). Moreover, C57 mice were injected with Saos‐2 cells to establish a subcutaneous xenograft model, and they were subsequently treated with three doses of BVP via intraperitoneal injection. The viability of the cells was examined by the Cell Counting Kit‐8 method. The apoptotic cells were assessed by flow cytometry and terminal deoxynucleotidyl transferase dUTP nick end labeling staining. The tumor volume and weight were monitored from day 3 through day 21 after the last injection. The expression of bax, bcl‐2, and cytochrome c (cyt c) mRNA was detected by a real‐time polymerase chain reaction. The protein levels of bax, bcl‐2, cyt c, caspase 3, and caspase 9 were evaluated by Western blot. The expression and distribution of bcl‐2 and bax in tissues were detected by immunohistochemistry. Compared with the control group, BVP treatment inhibited cell proliferation and induced apoptosis of Saos‐2 cells in vitro. Consistently, treatment of mice bearing transplanted tumors with BVP suppressed the growth of OS tumors and promoted cell apoptosis; it also reduced tumor volume and weight. Mechanistically, BVP‐induced apoptosis was mediated by the mitochondria‐dependent pathway, as evidenced by the increased expression of bax and cyt c and the decreased expression of bcl‐2, as well as activation of caspase 9 and caspase 3 in vitro and in vitro. Collectively, BVP inhibits growth and promotes apoptosis of OS by activating the mitochondrial apoptosis pathway.

## 1 INTRODUCTION

Osteosarcoma (OS) is the most common orthopedic malignant tumor in adolescents. OS occurs in cartilage, bone tissue, and muscles, accounting for approximately 0.2% of all tumors,^[^ ^1^ ^]^ and it is more prevalent in men.^[^ ^2^ ^]^ It has a high metastasis rate and mortality, and poor conventional treatment.^[^ ^3^ ^]^ The incidence for OS is 0.3 per 100 000 people per year.^[^ ^4^ ^]^ OS has a high degree of malignancy and poor prognosis. Local and distant metastasis occur early, and the treatment effect is often unsatisfactory.^[^ ^5^ ^]^ The survival rate of patients with OS is low and the treatment has not achieved breakthroughs.^[^ ^6^ ^]^ The overall 5‐year survival rate for patients with metastatic or relapsed OS has been around 20% for the past 30 years, and relapse rates have remained high at approximately 35%.^[^ ^4^ ^]^ The treatment effect of OS is not ideal. Due to overreliance on chemotherapy drugs and drug abuse, it is common for patients to have drug resistance in OS chemotherapy.^[^ ^7^ ^]^ The surgical treatments play a significant role in the treatment of OS. However, studies have shown that surgical trauma can cause a stress response, neuroendocrine disorders, and massive release of various inflammatory factors and an imbalance of the immune system, which results in the possibility of postoperative tumor metastasis and recurrence.^[^ ^8^ ^]^ Therefore, it is important to find a safe and effective way to treat the OS.

Apoptosis plays an important role in the occurrence and treatment of malignant tumors. Apoptosis induction of tumor cells has been an important way to treat tumors.^[^ ^9^ ^]^ Studies have shown that the growth of OS can be inhibited by promoting the mitochondrial apoptotic pathway.^[^ ^10^ ^]^ Mitochondrial pathway‐mediated apoptosis is one of the important forms of cell death.^[^ ^11^ ^]^ After the mitochondrial membrane permeability changes due to drug reasons, the proapoptotic proteins in the mitochondria including mitochondrial cytochrome c (cyt c) are released into the cytoplasm. The release of cyt c depends on the mutual regulation of bcl‐2 and bax. When cyt c is placed in the cytoplasm, it activates the underlying caspase 3 and caspase 9 zymogen. The cleaved caspase 3 (c‐caspase 3) and cleaved caspase 9 (c‐caspase 9) would act on different downstream targets as effectors and finally promote cell apoptosis.^[^ ^12^ ^]^

Natural products play a critical role in the discovery and development of numerous drugs for the treatment of various types of deadly diseases, like breast cancer by regulating autophagy and AMP‐activated protein kinase/mammalian target of rapamycin pathway^[^ ^13^ ^]^ and head and neck squamous cell carcinoma by immunomodulatory treatment.^[^ ^14^, ^15^ ^]^ Breviscapine (BVP) is the main extract of *Erigeron breviscapus*, which is a flavonoid compound. It exhibits strong pharmacological effects such as anti‐inflammation,^[^ ^16^ ^]^ antioxidation,^[^ ^16^ ^]^ antiplatelet aggregation,^[^ ^17^ ^]^ circulation improvement,^[^ ^18^ ^]^ and neuroprotection.^[^ ^19^ ^]^ In addition, BVP exhibits significant antitumor effects. A large number of studies have shown that BVP has different degrees of inhibition against liver cancer,^[^ ^20^ ^]^ prostate cancer,^[^ ^21^ ^]^ and lung cancer.^[^ ^22^ ^]^ However, it has yet not been reported whether BVP has an inhibitory effect on OS. Therefore, this study investigated whether BVP could inhibit the OS through inducing the mitochondrial apoptosis pathway.

## 2 MATERIALS AND METHODS

### 2.1 Regents

The human OS Saos‐2 cell line was purchased from the China Center for Type Culture Collection (Wuhan University). BVP was purchased from Hunan Hengsheng Pharmaceutical Co, Ltd. Dulbecco's modified Eagle's medium (DMEM) and fetal bovine serum (FBS) were purchased from Gibco. Bcl‐2, bax, cyt c, caspase 3, c‐caspase 3, caspase 9, c‐caspase 9, and glyceraldehyde‐3‐phosphate dehydrogenase (GAPDH) were purchased from Wuhan Proteintech Biotechnology Co, Ltd (Wuhan, China). The Apoptosis Kit was purchased from BD (CA). TRIzol and qRT‐PCR kits were purchased from TAKARA Biological Engineering Co, Ltd (Dalian, China).

### 2.2 Cell culture and treatment

Human OS Saos‐2 cells were cultured in DMEM containing 10% FBS in a 37°C, 5% CO_2_ incubator. We first used dimethyl sulfoxide to dissolve BVP into a 20‐μg/μL stock solution. Before using BVP to intervene cells, the stock solution was diluted with DMEM complete medium containing 10% FBS to 80, 160, and 320 μg/mL, and then added them to the cells. The cells were divided into control group, BVP low‐concentration group (80 μg/mL), BVP medium‐concentration group (160 μg/mL), and BVP high‐concentration group (320 μg/mL). The concentration of BVP for the treatment of cells was referenced from the published article.^[^ ^20^ ^]^ Saos‐2 cells in the logarithmic growth phase were transferred to a 6‐well plate or 96‐well plate. Then the cells in the treatment group were treated accordingly. The morphology of the cells was observed by an inverted phase‐contrast microscope.

### 2.3 Animal experiment

Thirty‐two male C57BL/6 mice (6‐8‐week‐old, weighing 18‐22 g) were purchased from the Hubei Provincial Health and Wellness Committee Animal Center. The mice were equally divided into four groups: control group, BVP low‐concentration group (7.5 mg/kg), BVP medium‐concentration group (15 mg/kg), and BVP high‐concentration group (30 mg/kg). The concentration of BVP for the treatment of mice was referenced from the published article.^[^ ^16^ ^]^ The BVP‐treated mice were inoculated with OS cells, Saos‐2. The Saos‐2 cells in the logarithmic growth phase were harvested. The number of cells was adjusted to 5 × 10^5^ cells/mL. Each mouse was inoculated in the forelimb to establish a subcutaneous transplanted tumor. Then the mice were injected with different BVP concentrations by the intraperitoneal administration. Intraperitoneal injection was conducted once every day for a total of 21 days. On the second day after the last injection, the mice were killed and the tumor volume and weight were measured.

### 2.4 Cell viability

When the cells were exponentially increasing, 96‐well plates were seeded. In total, 5000 cells/100 μL of medium per well were seeded. After the cells were attached, the cells were exchanged and treated. After 24 hours, 10‐μL Cell Counting Kit‐8 was added to each well. The medium was aspirated after 2 hr, and the optical density value at 570 nm was measured by a microplate reader.

### 2.5 Apoptosis assessment

The cells were seeded at a cell density of 10^5^/mL, and each group was given the corresponding treatment after the cells were attached. After 24 hours of treatment, the supernatant was discarded and the cells were resuspended by adding 500 μL of binding buffer. Subsequently, 5 μL of Annexin V‐FITC and 5 μL of propidium iodide were added, respectively, and reacted at room temperature for 15 minutes. Flow cytometry was used to detect apoptosis.

### 2.6 Quantitative real‐time polymerase chain reaction

Total RNA in cells and tissues was extracted according to the TRIzol instructions. The RNA was then reverse‐transcribed into complementary DNA (cDNA) according to the instructions of the Reverse Transcription Kit. The reverse transcription conditions were as follows: 37°C for 15 minutes, 85°C for 5 seconds, and 4°C for 10 minutes. Quantitative real‐time polymerase chain reaction (PCR) was carried out on a PCR machine using the reverse transcription product cDNA as a template. The reaction conditions were as follows: denaturation at 95°C for 5 seconds, 60°C for 10 seconds, and 40 cycles. The relative quantitative analysis was carried out by the $2^{-\Delta \Delta C_{t}}$ method. All the primer sequences of genes are listed in Table 1.

**Table 1:** The primer sequence of genes

Genes	Primer	Sequences (5′‐3′)
bcl‐2	Forward	GGTGGGGTCATGTGTGTGG
	Reverse	CGGTTCAGGTACTCAGTCATCC
bax	Forward	CCCGAGAGGTCTTTTTCCGAG
	Reverse	CCAGCCCATGATGGTTCTGAT
cyt c	Forward	CTTTGGGCGGAAGACAGGTC
	Reverse	TTATTGGCGGCTGTGTAAGAG
GAPDH	Forward	GGTCGGAGTCAACGGATTTG
	reverse	GGAAGATGGTGATGGGATTTC

Abbreviations: cyt c, cytochrome c; GAPDH, glyceraldehyde‐3‐phosphate dehydrogenase.

### 2.7 Western blot assay

Total protein in cells and animal tissues was extracted by the radioimmunoprecipitation assay lysis buffer. The protein concentration was determined by using the BCA Protein Concentration Assay Kit. Sodium dodecyl sulfate‐polyacrylamide gel electrophoresis (SDS‐PAGE) gel was used for electrophoresis. Then the protein in SDS‐PAGE gel was transferred into a polyvinylidene fluoride membrane. The membrane was then blocked by 5% skim milk for 1 hour. The corresponding bcl‐2 (1:1000), bax (1:1000), cyt C (1:1000), caspase 3 (1:1000), c‐caspase 3 (1:1000), caspase 9 (1:1000), c‐caspase 9 (1:1000), and GAPDH (1:1000) primary antibodies were incubated overnight at 4°C on a horizontal shaking table. The next day, the corresponding secondary antibody was incubated for 1 hour at room temperature. ECL luminous solution was added, and exposure analysis was performed on a chemiluminescence imager.

### 2.8 Immunohistochemical detection

The immunohistochemical paraffin was embedded in 4‐μm serial sections and placed on a glass slide. The endogenous peroxidase activity was eliminated by microwave treatment in an acidic environment, and 3% H_2_O_2_ and goat serum were added to the sections for 20 minutes to block the nonspecific protein. Then bax (1:200) and bcl‐2 (1:200) primary antibody dilution were performed at 4°C overnight. The human secondary antibody was added after rewarming. The DAB solution was added under a microscope to observe the color. Finally, hematoxylin counterstaining, alcohol dehydration and formaldehyde transparent sealing were conducted. The sections were observed and photographed under an inverted microscope.

### 2.9 Terminal deoxynucleotidyl transferase dUTP nick end labeling staining

Different groups of OS tissues were sectioned. According to the TUNEL Kit instructions, the tissues were stained at 37°C for 30 min, protected by 4′,6‐diamidino‐2‐phenylindole for 60 seconds. Then they were washed with phosphate‐buffered saline and blotted by filter paper. The fluorescent antiquenching agent was added. The sections were sealed with transparent nail polish. The conventional labeling index is equal to the number of positive cells in each visual field/all cells in the visual field. Three visual fields were selected. All cells in each visual field were counted, and the marker index of each visual field was counted. The apoptosis index of each group was equal to the average of each visual field marker index.^[^ ^23^ ^]^

### 2.10 Statistical analysis

All data are expressed as the mean ± SD values. Measurement data were analyzed by SPSS 13.0 statistical software. The differences among three or more groups were analyzed using a one‐way analysis of variance, followed by Bonferroni's post hoc test. *P* value less than .05 was statistically significant.

## 3 RESULTS

### 3.1 BVP treatment decreased the viability of Saos‐2 cells

**Figure 1:**
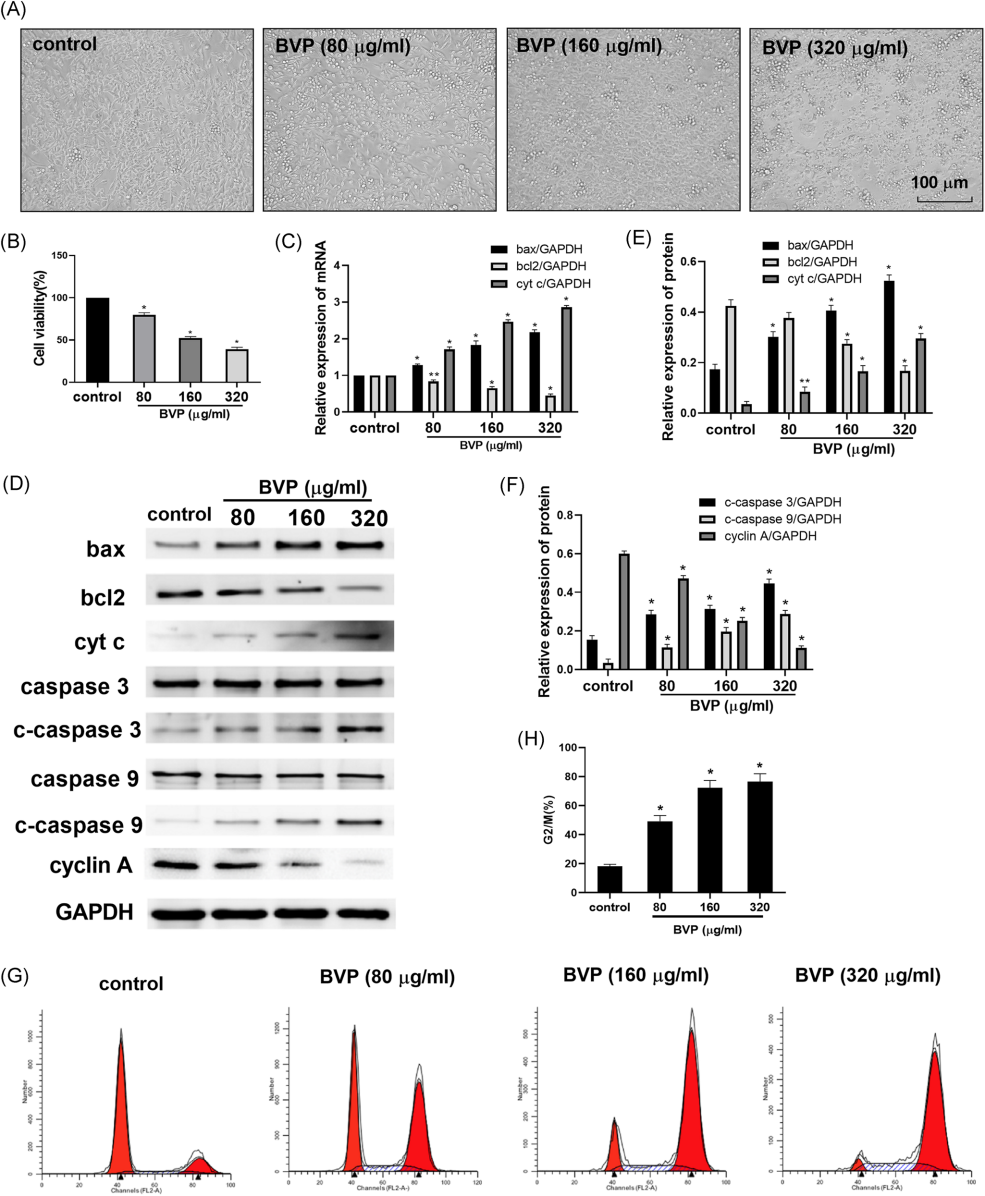
The effect of breviscapine (BVP) on cell morphology and viability. A, The morphology of the Sacos‐1 cells was observed under a light microscope. B, The Sacos‐1 cell viability was detected by Cell Counting Kit‐8 kit. C, The bax, cytochrome c (cyt c), and bcl‐2 mRNA levels were detected by a real‐time polymerase chain reaction. D‐F, The bax, cyt c, bcl‐2, caspase 3, cleaved caspase 3 (c‐caspase 3), caspase 9, cleaved caspase 9 (c‐caspase 9), and cyclin A protein levels were detected by Western blot. G,H, The cell cycle was detected by flow cytometry. N = 3. **P < .05 (analysis of variance [ANOVA]), compared with the control group. *P < .01 (ANOVA), compared with the control group. GAPDH, glyceraldehyde‐3‐phosphate dehydrogenase

To investigate the effect of BVP on Saos‐2 cells, we first examined the morphology and viability of cells. As shown in Figure 1A, control Sacos‐1 cells were round and attached to the cell culture plate under a light microscope. However, after treatment with BVP, the morphology of the cells was changed and they became smaller. Some of the cells were detached at lower concentrations; however, this phenomenon was more pronounced and cells were aggregated into clumps with the increase in BVP concentrations. Consistent with this, treatment of cells with BVP resulted in a dose‐dependent inhibition of cell growth as compared with the control group (*P* < .01; Figure 1B).

### 3.2 BVP treatment activated mitochondrial apoptosis pathway in Saos‐2 cells

As shown in Figure 1C, compared with the control group, the expression of bax and cyt c mRNA in BVP‐treated groups was dose‐dependently increased (*P* < .01). The expression of bcl‐2 in the 80‐μg/mL BVP‐treated group was significantly decreased (*P* < .05), and the mRNA expression of the BVP group was significantly decreased at 160‐ and 320‐μg/mL concentrations (*P* < .01). As shown in Figure 1D‐F, the expression of bcl‐2 protein in the 160‐ and 320‐μg/mL BVP‐treated group was significantly lower than that in the control group (*P* < .01). The bax, c‐caspase 3, and c‐caspase 9 protein levels in the three treatment groups were significantly increased (*P* < .01). The level of cyt c protein in the 80‐μg/mL BVP‐treated group was increased (*P* < .01). The expression of cyt c protein in the cells treated with 160‐ and 320‐μg/mL BVP was significantly increased (*P* < .01). However, there was no difference for caspase 3 and caspase 9 between groups.

### 3.3 BVP treatment inhibited division and induced apoptosis in Saos‐2 cells

**Figure 2:**
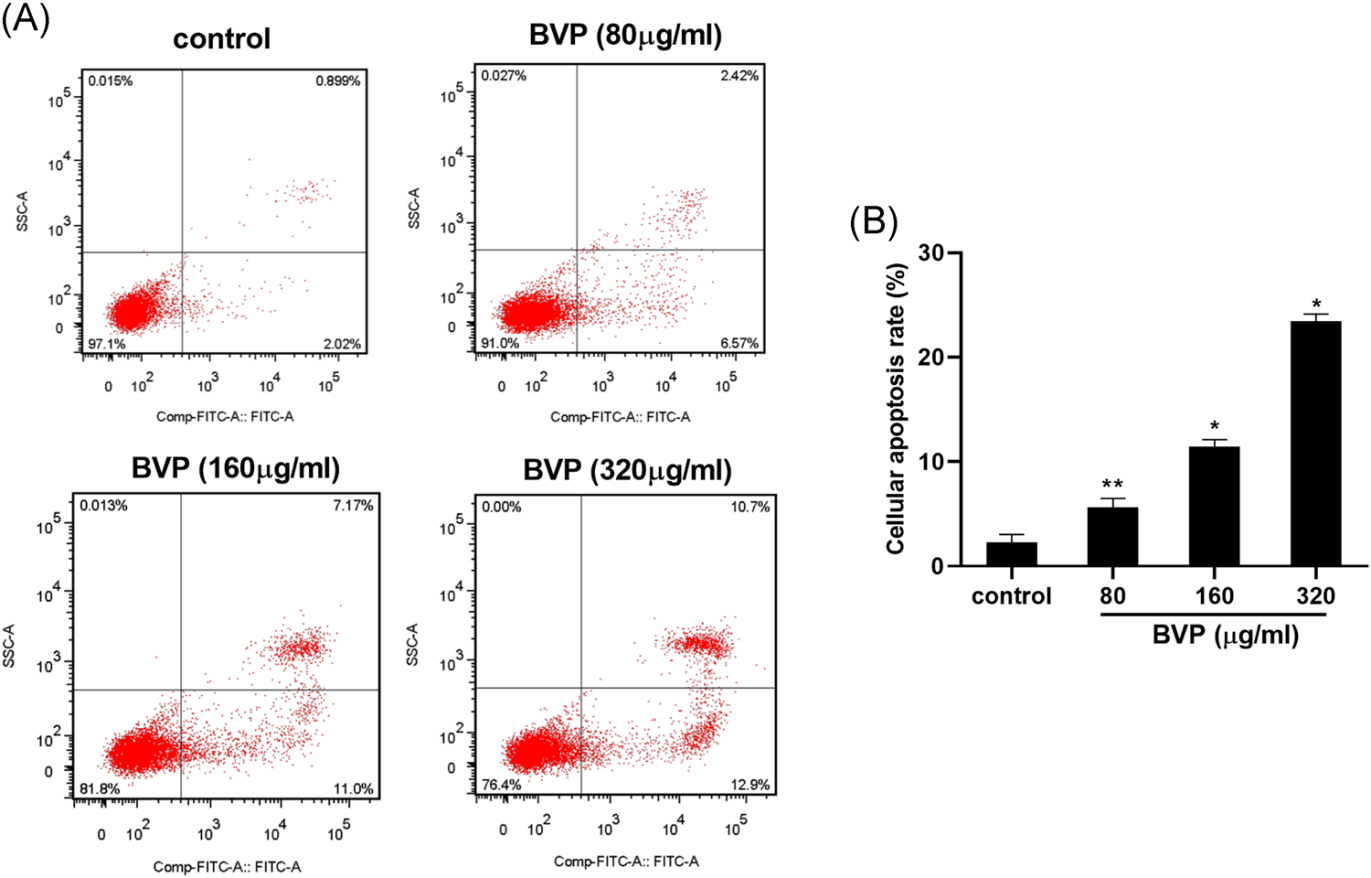
The effect of breviscapine on the apoptosis rate of Sacos‐1. A,B, The Sacos‐1 cell apoptosis rate was detected by flow cytometry. N = 3. **P < .05 (analysis of variance [ANOVA]), compared with the control group. *P < .01 (ANOVA), compared with the control group. BVP, breviscapine

To evaluate whether BVP could inhibit cell division and induce apoptosis, Saos‐2 cells were treated with various concentrations of BVP for 24 hours and the induction of apoptosis was analyzed by flow cytometry. As shown in Figures 1D and 1H, compared with the control group, the protein level of cyclin A in BVP‐treated groups was dose‐dependently decreased (*P* < .01). The number of cells in the G2/M phase in the BVP‐treated group was significantly increased (*P* < .05). BVP treatment significantly increased the percentages of apoptotic cells in a dose‐dependent fashion, with higher concentrations being more effective (*P* < .05; Figure 2A).

### 3.4 BVP treatment reduced the volume and weight of transplanted tumors in mice

**Figure 3:**
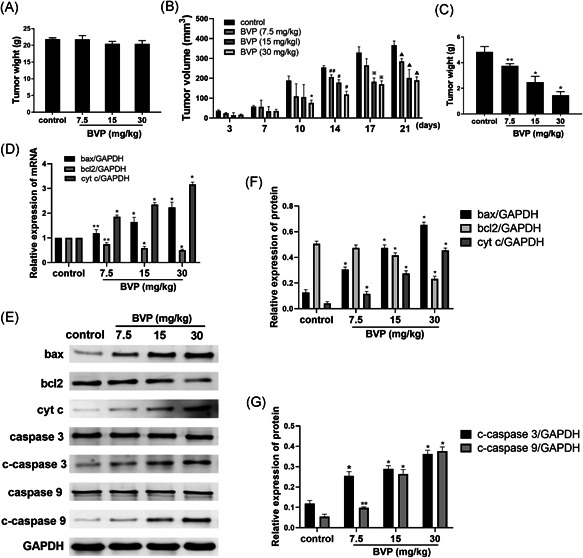
A, The effect of breviscapine on the tumor. B, The tumor volume on days 3, 7, 10, 14, 17, and 21 in different groups was detected. *P < .01, compared with the control group on day 10. ^##^ *P* < .05 (analysis of variance [ANOVA]), compared with the control group on day 14. ^#^ *P* < .01 (ANOVA), compared with the control group on day 14. ^※^ *P* < .01, compared with the control group on day 17. ^▲^ *P* < .01 (ANOVA), compared with the control group on day 21. C, The tumor weight on day 21 in different groups was detected. D, The bax, cytochrome c (cyt c), and bcl‐2 mRNA levels were detected by a real‐time polymerase chain reaction. E‐G, The bax, cyt c, bcl‐2, caspase 3, cleaved caspase 3 (c‐caspase 3), caspase 9, and cleaved caspase 9 (c‐caspase 9) protein levels were detected by Western blot. N = 3. **P < .05 (ANOVA), compared with the control group. *P < .01 (ANOVA), compared with the control group. GAPDH, glyceraldehyde‐3‐phosphate dehydrogenase

For the in vivo experiments, we first measured the weight of each group of mice to assess the toxicity of BVP to normal mice. As shown in Figure 3A, the weight in BVP groups showed no significant difference with the control group. As shown in Figure 3B, on the third day and the seventh day after transplantation, there was no difference in the size of the transplanted tumors of each group. On the first day after transplantation, the size of the tumor in the 30‐mg/kg BVP‐treated group was significantly reduced as compared with the control group (*P* < .01). On the 14th day after transplantation, the size of the tumor in the 7.5‐μg/mL BVP‐treated group was reduced as compared with the control group (*P* < .05), and the size of the tumor in the 15‐ and 30‐mg/kg BVP‐treated groups was significantly reduced (*P* < .01). On the 17th day after transplantation, the size of the tumors in the 15‐ and 30‐mg/kg BVP‐treated groups was significantly reduced as compared with the control group (*P* < .01). On the 21st day after transplantation, the tumor size of the three groups of BVP‐treated groups was significantly reduced as compared with the control group (*P* < .01). Subsequently, on the 21st day, the tumor suppressor of each group of mice was taken out. As shown in Figure 3C, the weight of the 7.5‐mg/kg BVP‐treated group was reduced as compared with the control group (*P* < .05), and the weight of the 15‐ and 30‐mg/kg BVP‐treated groups was significantly reduced (*P* < .01).

### 3.5 BVP treatment activated the mitochondrial apoptosis pathway in transplanted tumors

As shown in Figure 3D, compared with the control group, the expression of bax mRNA in the 7.5‐mg/kg BVP‐treated group was increased (*P* < .05). The expression of bcl‐2 mRNA in the 7.5‐mg/kg BVP‐treated group was decreased (*P* < .05). The expression of bax mRNA in the 15‐ and 30‐mg/kg BVP‐treated groups was significantly increased (*P* < .01). The expression of bcl‐2 mRNA in the 15‐ and 30‐mg/kg BVP‐treated groups was significantly decreased (*P* < .01). The cyt c mRNA levels in the three BVP‐treated groups were significantly increased (*P* < .01). As shown in Figure 3E‐G, the expression of bcl‐2 protein in the 15‐ and 30‐mg/kg BVP‐treated groups was significantly lower than that in the control group (*P* < .01). The bax, cyt c, and c‐caspase 3 protein levels of the three treatment groups were significantly increased (*P* < .01). The level of cyt c protein in the 7.5‐mg/kg BVP‐treated group was increased (*P* < .01). The expression of c‐caspase 9 protein in the cells treated with 15‐ and 30‐mg/kg BVP was significantly increased (*P* < .01). However, there was no difference for caspase 3 and caspase 9 between groups.

### 3.6 BVP treatment induced apoptosis and inhibited division of transplanted tumor cells in mice

**Figure 4:**
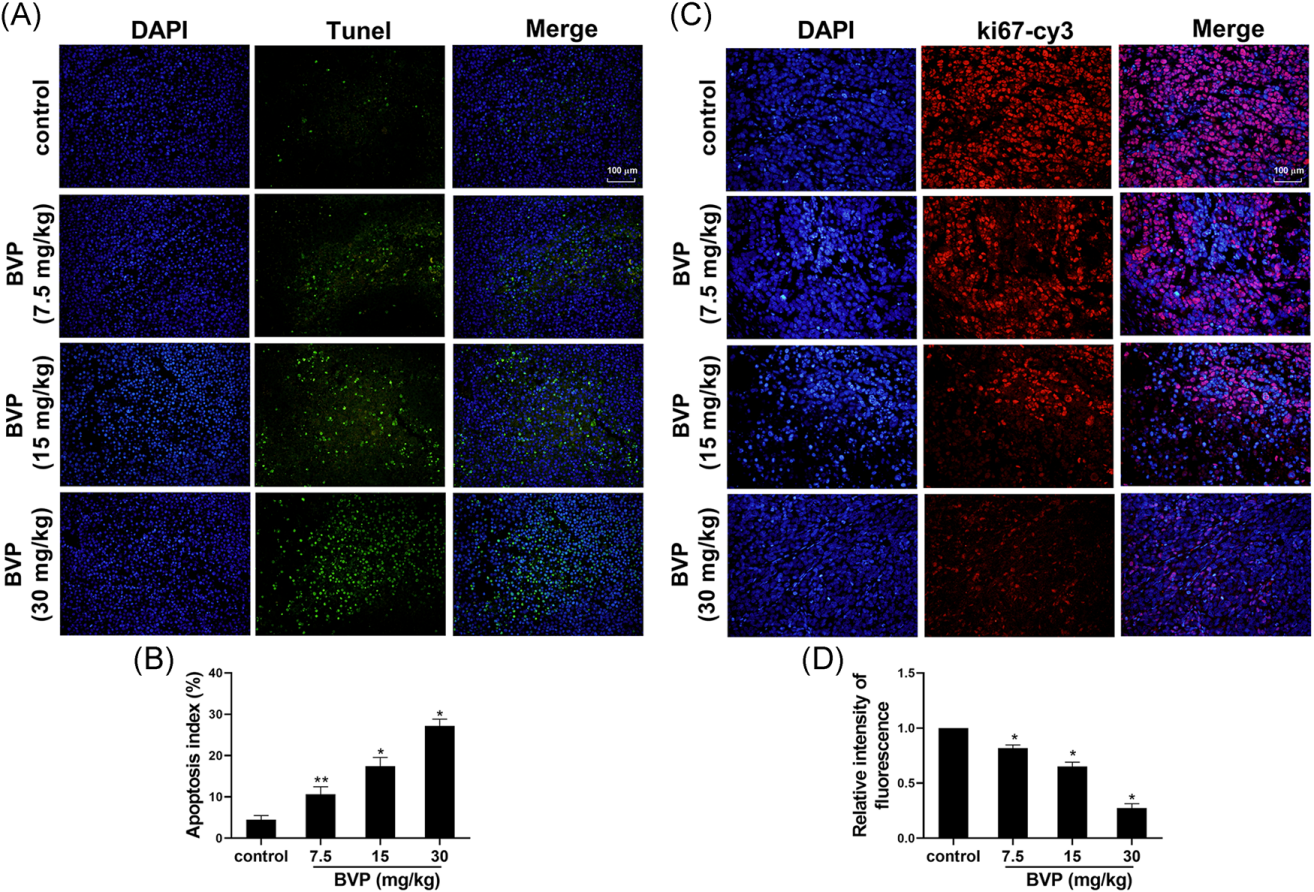
The effect of breviscapine on the apoptosis rate of tumor cells in the tumor. A,B, Apoptotic myeloma cells in the tumor were detected by terminal deoxynucleotidyl transferase dUTP nick end labeling staining. C,D, The ki67 protein in tumor tissues was detected by immunofluorescence. N = 3. **P < .05 (analysis of variance [ANOVA]), compared with the control group. *P < .01 (ANOVA), compared with the control group. DAPI, 4′,6‐diamidino‐2‐phenylindole

As shown in Figure 4A,B, the level of terminal deoxynucleotidyl transferase dUTP nick end labeling (TUNEL)‐stained cells in the 7.5‐mg/kg treatment group were higher than that in the control group (*P* < .05). The level of TUNEL‐stained cells in 15‐ and 30‐mg/kg treatment groups was significantly increased (*P* < .01). As shown in Figure 4C,D, compared with the control group, the protein level of ki67 in the BVP‐treated group was significantly decreased (*P* < .05).

### 3.7 The impact of BVP treatment on bax and bcl‐2 in transplanted tumors

**Figure 5:**
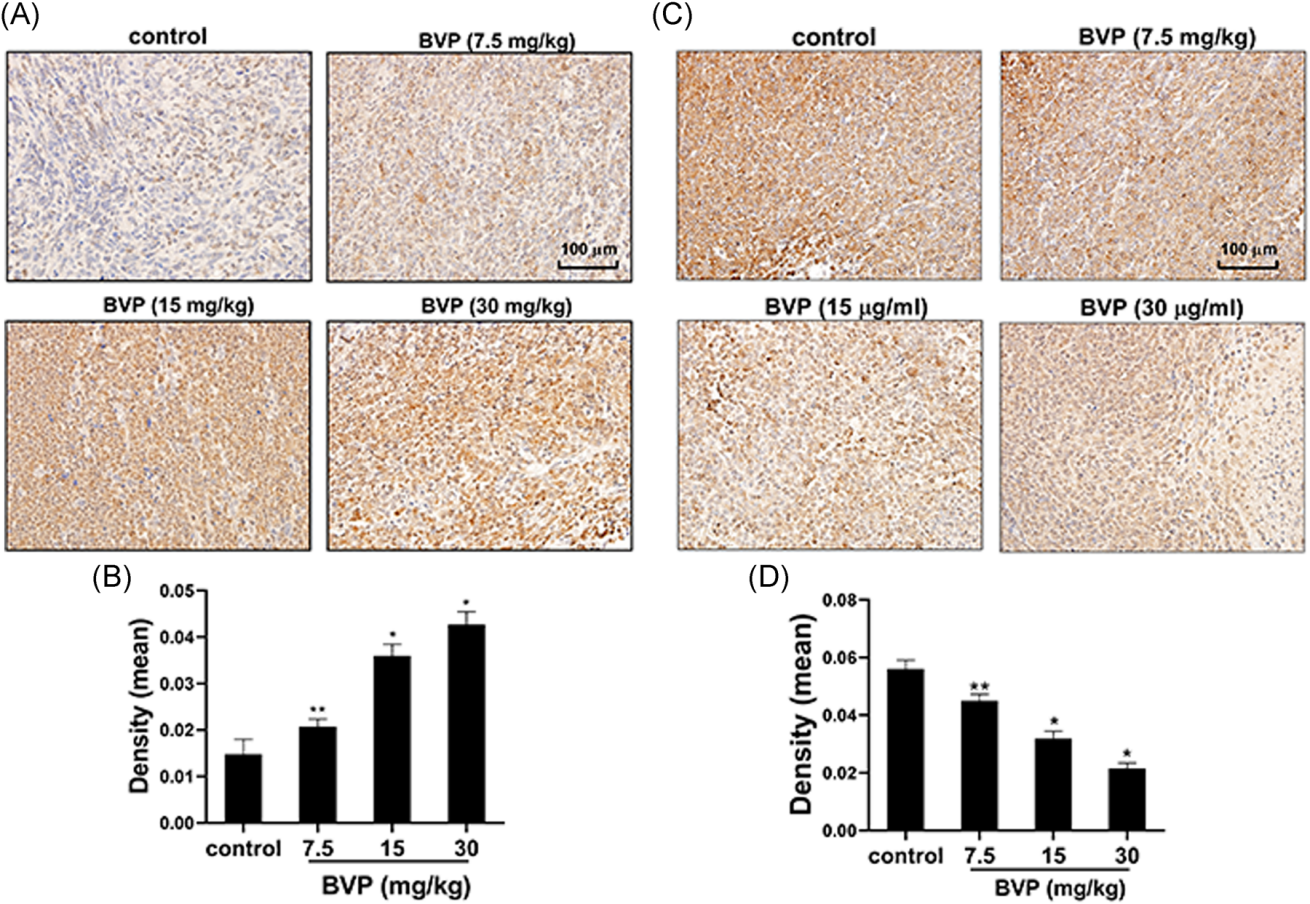
The effect of breviscapine on bax and bcl‐2 molecules in the tumor. A,B, The distribution and expression of bax protein in tumors were detected by immunohistochemistry. C,D, The distribution and expression of bax protein in tumors were detected by immunohistochemistry. N = 3. **P < .05 (analysis of variance [ANOVA]), compared with the control group. *P < .01 (ANOVA), compared with the control group. BVP, breviscapine

As shown in Figure 5A,B, the expression of bax protein in the 7.5‐mg/kg BVP‐treated group was increased than that in the control group (*P* < .05). The expression of bax protein in the 15‐ and 30‐mg/kg BVP‐treated groups was significantly increased than that in the control group (*P* < .01). As shown in Figure 5C,D, the expression of bcl‐2 protein in the 7.5‐mg/kg BVP‐treated group was lower than that in the control group (*P* < .05). The expression of bcl‐2 protein in the 15‐ and 30‐mg/kg BVP‐treated groups was significantly decreased than that in the control group (*P* < .01).

## 4 DISCUSSION

OS is an invasive tumor of mesenchymal stem cell‐derived bone mass, which represents the most common primary malignant tumor.^[^ ^24^ ^]^ According to the metastatic nature of the tumor, it is divided into the nonmetastatic and metastatic OS. For nonmetastatic OS, the patient's 5‐year survival rate can reach 40% to 75% after surgical resection and adjuvant chemotherapy; however, about 20% of OS patients have a metastatic OS with local infiltration and distant metastasis. Although there are many modern therapies, including chemotherapy, radiotherapy, and comprehensive surgery, which are commonly used in clinical practice, the morbidity and mortality of patients with OS are still high.^[^ ^25^, ^26^ ^]^ Conventional chemoradiotherapy is likely to cause serious side effects, often leading to drug resistance. In addition, due to the rapid growth of OS in the early stage, it easily infiltrates and metastasizes to other tissues, and there is still a large recurrence probability after surgical resection. The pathogenesis of OS is not yet clear, and it is highly heterogeneous in its manifestation, which makes targeted therapy quite difficult.^[^ ^27^, ^28^ ^]^ Therefore, finding a safe, low‐toxic, and highly targeted drug that greatly improves the survival rate of patients is a major goal in the treatment of OS. At present, analogs such as BVP, paclitaxel, and vincristine extracted from natural plants have been widely used in the treatment of tumors, and the curative effect is exact.

Apoptosis is regulated by a variety of signaling proteins. The well‐known apoptosis pathways include the death receptor pathway and the mitochondrial apoptosis pathway. The mitochondrial apoptosis pathway is more likely to induce apoptosis via the caspase cascade.^[^ ^29^ ^]^ Mitochondrial pathway‐induced apoptosis mainly includes the following aspects: the mitochondrial membrane permeability transition pore is activated and then cyt c releases from the mitochondrial matrix into the cytoplasm. The released cyt c formats with dATP dimer binding to apaf‐1. Apaf‐1 activates procaspase 9 into c‐caspase 9. Then c‐caspase 9 activates caspase 3 precursor into c‐caspase 3, which could induce cell apoptosis. The release of cyt c depends on the mutual regulation of bcl‐2 and bax. Bcl‐2 and bax are located on the mitochondrial membrane. The imbalance between the two can activate the mitochondrial permeability transition pore and change the permeability of the mitochondrial membrane leading to cells. The release of cyt c induces an apoptotic response.^[^ ^30^ ^]^

**Figure 6:**
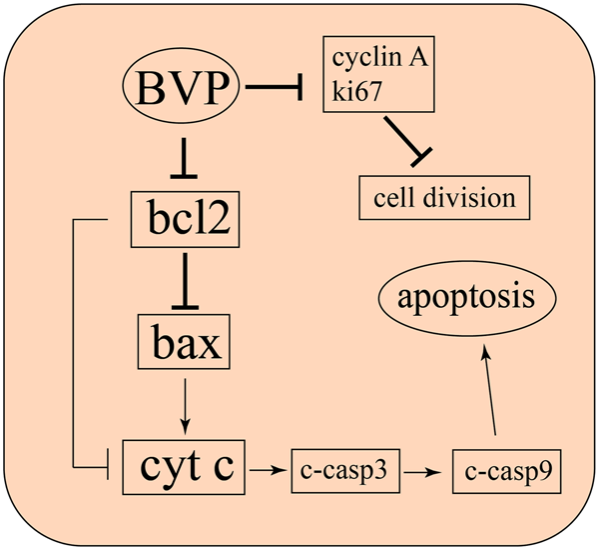
Breviscapine (BVP) inhibits growth and promotes apoptosis of osteosarcoma by activating the mitochondrial apoptosis pathway. BVP‐induced apoptosis was mediated by the mitochondria‐dependent pathway, as evidenced by the increased expression of bax and cytochrome c (cyt c) and the decreased expression of bcl‐2, as well as activation of caspase 9 and caspase 3 in vitro and in vitro. Meanwhile, BVP could inhibit the division of osteosarcoma cells through inhibiting the expression of cyclin A and ki67. c‐caspase 3, cleaved caspase 3; c‐caspase 9, cleaved caspase 9

In this experiment, the human OS cell line Saos‐2 was first treated with BVP. After the treatment of BVP, some of the tumor cells became smaller and were detached. The cell viability was decreased. Compared with control cells, the expression of bax, cyt c, c‐caspase 3, and c‐caspase 9 in BVP‐treated groups was significantly increased, whereas the level of bcl‐2 was decreased. The apoptotic cells in BVP‐treated groups were increased. Moreover, BVP could inhibit cell division by inhibiting the expression of cyclin A. In the in vivo experiment, the growth rate and size of the tumor were inhibited by BVP. The expression of bax, cyt c, c‐caspase 3, and c‐caspase 9 in BVP‐treated groups was significantly increased, whereas the level of bcl‐2 was decreased when compared with control mice. The expression of bax2, bcl‐2, and ki67 protein in OS was then detected by immunohistochemistry or immunofluorescence. The expression of bax protein was increased, whereas the bcl‐2 and ki67 protein was decreased in the BVP‐treated group than the control group. The apoptotic cells in the BVP‐treated group were also elevated. The experiment showed that the protein expression of bcl‐2 was decreased, whereas the protein expression of the proapoptotic gene bax was continuously increased. Therefore, it is speculated that bcl‐2 and bax are the main molecules that participate in BVP‐induced apoptosis (Figure 6).

In summary, we have shown that BVP promotes apoptosis of OS cells by activating the mitochondrial apoptosis pathway. To the best of the authors' knowledge, this study is the first to demonstrate that BVP possesses potent antiosteosarcoma activity in vitro and in vivo. Our findings provide a basis for OS clinical treatment. However, its security and in‐depth mechanism need to be further studied.

## CONFLICT OF INTERESTS

The authors declare that there are no conflict of interests.
